# Delayed definitive management of localized prostate cancer: what do we know?

**DOI:** 10.1038/s41391-024-00876-2

**Published:** 2024-08-11

**Authors:** Osama Mohamad, Yun Rose Li, Felix Feng, Julian C. Hong, Anthony Wong, Zakaria El Kouzi, Mohamed Shelan, Thomas Zilli, Peter Carroll, Mack Roach

**Affiliations:** 1 Department of Genito-urinary Radiation Oncology, MD Anderson Cancer Center, Houston, TX USA; 2 Department of Radiation Oncology, City of Hope Cancer center, Duarte, CA USA; 3 Department of Radiation Oncology, University of California San Francisco, San Francisco, CA USA; 4 Department of Urology, University of California San Francisco, San Francisco, CA USA; 5 Department of Radiation Oncology, Inselspital Bern, University of Bern, Bern, Switzerland; 6 Department of Radiation Oncology, Oncology Institute of Southern Switzerland, EOC, Bellinzona, Switzerland; 7 Facoltà di Scienze biomediche, Università della Svizzera italiana, Lugano, Switzerland

**Keywords:** Outcomes research, Urological cancer

## Abstract

Delays in the work-up and definitive management of patients with prostate cancer are common, with logistics of additional work-up after initial prostate biopsy, specialist referrals, and psychological reasons being the most common causes of delays. During the COVID-19 pandemic and the subsequent surges, timing of definitive care delivery with surgery or radiotherapy has become a topic of significant concern for patients with prostate cancer and their providers alike. In response, recommendations for the timing of definitive management of prostate cancer with radiotherapy and radical prostatectomy were published but without a detailed rationale for these recommendations. While the COVID-19 pandemic is behind us, patients are always asking the question: “When should I start radiation or undergo surgery?” In the absence of level I evidence specifically addressing this question, we will hereby present a narrative review to summarize the available data on the effect of treatment delays on oncologic outcomes for patients with localized prostate cancer from prospective and retrospective studies.

## Introduction

Delayed treatment of prostate cancer is a common occurrence in patients presenting with clinically localized disease [1, 2]. Frequently, such events are brought up in the context of planned vacations, business trips, logistics, psychosocial reasons, national disasters (e.g. floods, hurricanes, earth quakes), or mechanical failures (e.g. linear accelerators). In addition, for patients with low-risk disease, an intentional deferment of treatment (termed “active surveillance”) is a common and often preferred management strategy [3]. In 2020, the COVID-19 pandemic has brought acute attention to this matter and caused a substantial shift in the risk-to-benefit ratio associated with managing these patients forcing providers to critically rethink the merits of screening, biopsies, diagnostic work-ups, and initiation of treatment [4, 5].

Given the fact that the majority of men with prostate cancer are older than the age of 65 at the time of diagnosis and may have pre-existing health conditions, most are at a higher risk for other-cause mortality especially those presenting with favorable risk [6, 7]. These patients with low-risk disease can live many years without any intervention and death from prostate cancer mostly affect younger men with high-grade or advanced stage disease [8, 9]. Nevertheless, delaying treatment must be balanced against the risk of prostate cancer progression especially in patients presenting with high-grade disease or advanced stage. In all cases, it is extremely important to educate our patients and their families about the natural history of prostate cancer and reassure them that prostate cancer tends to progress relatively slowly in the vast majority of cases [10].

In response to the COVID-19 pandemic, recommendations for the definitive management of localized prostate cancer with radiotherapy and radical prostatectomy have been published but the evidence supporting the explicit impact of delays on outcomes was not included [11, 12]. The National Comprehensive Cancer Network (NCCN) has also published guidelines for the management and for early detection of prostate cancer during the COVID-19 pandemic [4, 5]. While there are no prospective phase III randomized clinical trials (RCTs) specifically designed to answer this question about acceptable delay periods, “evidence-based” recommendations concerning the impact of delayed definitive treatment can be extrapolated from a number of sources [13, 14]. The aim of this paper is thus to perform a narrative review of the literature on the available evidence of the impact of delays in the definitive treatment [using either radiotherapy (RT) or radical prostatectomy (RP)] of prostate cancer on oncologic outcomes. While the COVID-19 pandemic was the impetus of this review, we believe this topic remains of great importance given the prevalence of delays in prostate cancer.

## Methods

Three authors (MR, OM, YL) searched the literature via Pubmed (from January 2000 to June 2020) for full papers (not abstracts) with the following words: delayed (OR deferred OR expectant management) AND prostate (OR prostate cancer) AND surgery (OR radiation). We limited our search to papers with available median (or mean) delay and clear definition of comparison groups. When duplicated or updated analyses were available, we used the most recent manuscript if it included the required data elements. Notably, there are two general categories of data that we used to estimate the potential impact of delayed RP or RT. The first category comes from information that can be extrapolated from phase III RCTs which incorporated multiple treatments (most staggered in time relative to the start of the radical treatment, specifically RP or RT). The second comes from post-hoc retrospective studies evaluating the impact of delays measured from the time of biopsy until the time of definitive local treatment by RP or RT. Interpretation of the impact of delays from RCTs, which compared immediate versus delayed definitive treatment, is based on two major assumptions. If the trial was positive (i.e. demonstrated an improvement in the specified outcome for the immediate treatment arm as compared to the delayed arm), then the delay was too long. In this case, one must assume a safe delay must be shorter than the one used in that trial. In contrast, if the delay in treatment (RP or RT) resulted in a survival that was comparable to or better than the immediate treatment arm, then the delay was not excessive. Regarding the interpretation of both the RCTs and retrospective studies, due to the heterogeneity in duration of delays, patient selection, exclusions and practice patterns (e.g. earlier treatment of high-risk patients), we concluded that it would not be appropriate to attempt to do a meta-analysis to assess the impact of delays on outcomes of either sets of data. Similarly, in the absence of any RCT specifically designed to answer whether delayed definitive management of prostate cancer would affect oncologic outcomes, we did not perform a systematic review. Finally, we did not formally assess quality or bias due to the heterogeneity of the data included in these studies.

### Data from randomized trials

#### Delayed definitive radiation or definitive radical prostatectomy

We identified 26 RCTs in which a delay in definitive local treatment (RT or RP) occurred in one of the treatment arms [15–40] (Table 1). Eight RCTs compared delayed RT preceded by neoadjuvant androgen deprivation (ADT) hormonal therapy (NHT) to immediate RT, or compared shorter durations of NHT to longer durations. These trials are RTOG 8610, RTOG 9408, D’Amico (these 3 studies compared 2 months of NHT vs. immediate RT), Crook (8 vs. 3 months of NHT), ICORG 9701 (4 vs. 8 months of NHT), RTOG 9910 (7 months vs. 2 months of NHT), TROG 9601 (2 vs. 5 months of NHT vs. immediate RT), and Malone (4 months of NHT vs. immediate RT + ADT) [15–22] (Table 1). Taken together, these studies (four with a radiation alone control arm) suggested that for patients with intermediate- to high-risk prostate cancer, delaying RT up to 7–8 months was safe if preceded by NHT. In fact, patients in the immediate RT alone arms consistently demonstrated worse outcomes than in the delayed RT (NHT + RT arm), reflecting the therapeutic benefit of ADT. Similarly, there are at least 6 trials comparing neoadjuvant ADT prior to RP [23–28] (Table 1). Taken together, these studies demonstrated unequivocally that delays of up to 3–8 months prior to RP or RT are likely to be safe if preceded by NHT. However, in contrast to RT-based therapy, results from the RP-specific studies did not demonstrate an improved survival in the NHT + RP arms. Therefore, NHT prior to RP should not be routinely recommended. However, in the event that RP needs to be delayed in patients with high-risk profiles, NHT for at least 3 months can be safely used to delay the start of radical therapy with likely no discernible decrease in outcomes.

**Table 1:** Published randomized trials providing evidence regarding the impact of treatment delay of radical local therapy for prostate cancer.

No.	Author (year) [reference]	Study name, if available	Design	Delay per trial protocol	Median FU (yrs)	Treatment years (*N*)	Conclusions	Comments
**Neoadjuvant ADT before RT**								
1	D’Amico, 2004 [8]		RT vs ADT→RT	2 mo ADT	4.52	1995–2001 (*N* = 206)	Improved OS and PCSM with delay + ADT	Total ADT 6 mo; mostly IR, some HR
2	Roach, 2008 [9]	RTOG 8610	RT vs ADT→RT	2 mo ADT	12.5	1987–1991 (*N* = 456)	Improved PCSM, DM and BF with delay + ADT	Total ADT 2 mo neoadjuvant and concurrent (4 mo); bulky cT2-4 +/− LNs
3	Crook, 2009 [10]		3 mo ADT→RT vs 8 mo ADT→RT	3 vs 8 mo ADT	6.6	1995–2001 (*N* = 378)	Overall no difference in biochemical DFS	Included LR, IR and HR; Improved DFS in subset of HR on 8-mo arm
4	Armstrong, 2010 [11]	ICORG 97-01	4 mo ADT→RT vs 8 mo ADT→RT	4 vs 8 mo ADT	8.5	1997–2001 (*N* = 276)	No advantage for prolonged ADT in OS, PCSM, or bPFS	Mostly HR
5	Denham, 2011 [12]	TROG 96.01	RT vs ADT→RT	0, 2 or 5 mo ADT	10.6	1996–2000 (*N* = 818)	Improved DM and PCSM in 6 mo ADT group vs no ADT	Total ADT 0 vs 3 mo vs 6 mo; Mostly HR.
6	Jones, 2011 [13]	RTOG 9408	RT vs ADT→RT	2 mo ADT	9.1	1994–2001(*N* = 1979)	Improved OS and PCSM with delay + ADT	Total ADT 4 mo; included LR/IR; benefit of ADT mostly for IR.
7	Pisansky, 2015 [14]	RTOG 9910	2 mo ADT→RT vs 7 mo ADT→RT	2 vs 7 mo ADT	9.4	2000–2004 (*N* = 1489)	Similar OS and cause-specific survival	Mostly IR; both arms received 2 mo ADT during and after RT
8	Malone, 2020 [15]		ADT→RT vs ADT + RT	0 vs 4 mo ADT	12.2	2002–2012 (*N* = 432)	Similar bPFS and OS	Total ADT 6 mo in both arms; Mostly IR.
**Neoadjuvant ADT before RP**								
9	Van Poppel, 1995 [16]		RP vs estramustine→RP	1.5 mo estramustine	0.75	*N* = 130	Similar rate of PSA failure and no survival advantage	Improvement in rate of positive margins for cT2b but not cT3
10	Homma, 1997 [17]		RP vs ADT→RP	3 mo ADT	2	1993–1995 (*N* = 224)	Same rate of PSA failure and OS; Reduction of positive margins and positive LNs.	Clinical stage A2, B, and C; both groups received adjuvant ADT
11	Aus, 1998 [18]		RP vs ADT→RP	3 mo ADT	3.2	*N* = 122	Same rate of PSA failure and OS; reduced rates of positive margins	T1b-T3a
12	Klotz, 1999 [19]	CUOG	RP vs anti-androgen→RP	3 mo ADT	3	1993–1994 (*N* = 213)	Similar rate of PSA failure and no survival advantage; reduced rates of positive margins	T1b-T2c
13	Schulman, 2000 [20]		RP vs ADT→RP	3 mo ADT	4	1991–1995 (*N* = 402)	Similar rate of PSA failure and no survival advantage; reduced rates of positive margins	cT2-T3
14	Soloway, 2002 [21]	LDNPCSG	RP vs ADT→RP	3 mo ADT	5	1992–94 (*N* = 282)	Similar rate of PSA failure and no survival advantage; reduced rates of positive margins	cT2b
**ADT +/− RT**								
15	Widmark, 2009 [22]	SPCG-7	ADT vs ADT + RT	RT vs no radical treatment	7.6	1996–2002 (*N* = 875)	Improved PCSM with RT	Included locally advanced or localized HR
16	Warde, 2011 [23]		ADT vs ADT + RT	RT vs no radical treatment	6	1995–2005 (*N* = 1205)	Improved OS with RT	T3-4 or PSA > 40 or PSA > 20 and GS > 7
**RP vs. observation**								
17	Bill-Axelson, 2018 [24]	SPCG-4	RP vs “watchful waiting” (WW)	RP vs no treatment	23.6	1989–99 (*n* = 695)	RP had improved OS	At long follow-up, a mean of 2.9 years was gained with RP
18	Wilt, 2020 [25]	PIVOT	RP vs observation	RP vs no treatment	18.6	1994–2002 (*N* = 713)	RP had improved OS	At long follow-up, a mean of 1 years was gained with RP
19	Neal, 2020 [26]	PROTECT	RP vs RT vs “active surveillance (AS)”	RP/RT vs AS	7.7	1999–2009 (*N* = 2640)	AS worse PCSS and DM than any radical treatment with 2x rate of metastasis and 3-4x rate of disease progression	RP and RT had similar oncologic outcomes but different toxicity profiles. Median 7 years delay on AS arm.
**Delaying post-prostatectomy RT**								
20	Thompson, 2009 [27]	SWOG 8794	Adjuvant RT vs observation	Immediate vs no RT	12.6	1988–1997 (*N* = 425)	Improved PSA control, DM, and OS with adjuvant RT	pT3N0; 70/211 observation arm ultimately received RT
21	Wiegel, 2009 [28]	ARO 96-02	Adjuvant vs Salvage RT	<3 mo vs RT at PSA failure	5	1997–2004 (*N* = 385)	Improved bPFS, no difference in DM or OS with adjuvant RT	pT3N0, PSA undetectable at time of RT; RT initiated at time of PSA failure in observation arm
22	Bolla, 2012 [29]	EORTC 22911	Adjuvant vs Salvage RT	<4 mo vs RT at time of failure	10.6	1992–2001 (*N* = 1005)	Improved PSA control in adjuvant arm	pT3 or + margins; 218/503 in wait and see arm received salvage RT at median of 2.9 years
23	Hackman, 2019 [30]	Finnish Study	Adjuvant RT vs observation (salvage RT allowed)	Immediate vs no RT	9.3	2007–2012 (*N* = 250)	Improved bPFS, no difference in DM or OS with adjuvant RT	pT2 with positive margins or pT3a; 37/43 patients with biochemical recurrence in the observation group received salvage RT
24	Parker, 2020 [31]	RADICALS-RT	Adjuvant vs early Salvage RT	Immediate RT vs RT at PSA failure	4.9	2007–2016 (*N* = 1396)	No difference in bPFS between 2 arms	Increased urinary toxicity with adjuvant RT
25	Kneebone, 2020 [32]	TROG 08.03/ANZUP RAVES	Adjuvant vs early Salvage RT	Immediate RT vs RT at PSA failure	6.1	2009–2015 (*N* = 333)	No difference in bPFS between 2 arms	Increased urinary toxicity with adjuvant RT
26	Sargos, 2020 [33]	GETUG-AFU 17	Adjuvant vs early Salvage RT	Immediate RT vs RT at PSA failure	6.25	2008–2016 (*N* = 424)	No difference in event-free survival between 2 arms	Increased genitourinary toxicity and erectile dysfunction with adjuvant RT

*FU* follow-up, *N* sample size, *LR* low risk, *IR* intermediate risk, *HR* high risk, *PSA* prostate specific antigen, *GS* Gleason score, *ADT* androgen deprivation therapy, *RT* radiation therapy, *RP* radical prostatectomy, *PCSM* prostate cancer-specific mortality, *PCSS* prostate cancer-specific survival, *OS* overall survival, *DM* distant metastasis, *BF* biochemical failure, *bPFS* biochemical progression-free survival, *DFS* disease-free survival, *AS* active surveillance, *LN* lymph nodes, *cT* clinical T-stage, *pT* pathological T-stage.

#### Benefit of local interventions

Two major RCTs compared hormonal therapy alone vs hormonal therapy and RT and demonstrated that the addition of RT improved overall survival (OS) [29, 30]. Similarly, three studies comparing immediate RP vs observation (Swedish SPCG-4, PIVOT, and ProtecT) suggest a benefit with RP compared to observation [31–33]. Results from the ProtecT trial were recently published [41]. This study compared immediate “radical” treatment (RP or RT) to active monitoring (AM). Men enrolled on the AM arm remained on AM for a median of 7.7 years and had a slightly increased risk of clinical progression [which included metastatic disease, local progression, or initiation of ADT]. Ultimately, 55% of patients progressing to T3-4 disease subsequently underwent RP (20%) or RT (35%) [41]. Pooled estimates of “radical” local treatment showed reduced incidence of prostate cancer deaths, metastatic disease, and the onset of ADT. “Radical” treatment was also associated with a reduction in prostate cancer mortality when compared to men either randomized to AM or who choose AM off study (although the number of deaths was relatively small). Notably, patients on the ProtecT trial had very favorable disease with a median PSA of ∼4.5 ng/ml, 75–80% had Gleason grade group 1, and with 2/3^rd^ low-risk disease. Thus, based on the data from this trial, delayed definitive local therapy should probably be less than 7 years to be safe (for patients with favorable-risk disease). It is noteworthy to mention that the benefits of radical intervention are less for older patients with screen-detected low-grade prostate cancer. Since the results associated with either RP or RT were similar, it is also reasonable to assume that similar durations of delays would apply to both equally for this favorable group of patients. These updated findings are consistent with those of the updated PIVOT trial [32].

#### Delayed post-operative radiotherapy

In the post-operative setting after RP, three “older” phase III trials compared immediate (“adjuvant”) post-operative RT to observation with delayed RT if necessary (i.e. “salvage” RT at time of clinical or biochemical failure at the discretion of treating physician) in patients with positive margins or pT3 disease [34–36]. In addition, a Finnish trial compared “early adjuvant” RT vs observation with salvage RT given to most patient with biochemical failure in the observation arm [37] (Table 1). Moreover, three contemporary trials (“RAVES”, “RADICALS”, and “GETUG-AFU 17) compared “early salvage” (a short delay until PSA 0.1-0.2) to immediate (adjuvant) RT [38–40] and have been recently presented, along with a planned meta-analysis [42]. While the SWOG 8794 study showed improved OS with adjuvant RT compared to observation, these RCTs suggest that post-operative RT can be safely delayed until PSA failure occurs (PSA 0.1-0.2). However, whether RT should be delayed in patients with higher risk pathologic/genomic features (especially seminal vesicle involvement, lymph node involvement, Gleason score 8-10, and/or high-risk genomics, etc.) remains to be answered. Of note, data from randomized studies suggests that salvage RT at earlier pre-RT PSA improves outcomes (RTOG 9601, RTOG 0534, and GETUG-AFU 16) [43–45] and the role of NHT to delay RT in that setting is less well understood.

Regarding the available data from multiple phase III RCTs, several conclusions could be drawn concerning the impact of a definitive treatment timing delay on the outcomes of local intervention for localized prostate cancer (RP or RT). First, for patients with intermediate- and high-risk prostate cancer, NHT can safely allow a delay of up to 8 months without affecting outcomes. Patients with more favorable disease could potentially be delayed on AS or AM for 7 years or less. In the post-operative setting, early adjuvant RT could be omitted with PSA follow-up to allow for early salvage RT. However, delaying RT in patients with biochemical recurrence may not be advisable but a period of NHT of at least 2 months is reasonable.

### Post-hoc retrospective series

We identified 39 retrospective studies which investigated the impact of delay, as measured from time of biopsy to definitive treatment (RT or RP), on prostate cancer outcomes including adverse pathologies after RP (See Appendix for references). The total number of patients was 1,164,164, including 1 SEER and 3 NCDB studies which alone contributed 1,107,404 patients. Four studies were restricted to patients treated with RT alone (*n* = 6870), 32 studies included patients treated with RP (*n* = 209,515), and three studies included both RT and RP patients (*n* = 947,779). Among the RT only studies, three explicitly excluded patients receiving ADT (Nguyen et al., Kwan et al., and Andrews el al.). For all patients in the 39 studies identified, the median delay (or mean when median was not available) generally appeared to be relatively short, and averaged approximately 3.4 months (approximate range 1.5–9.9 months). A major challenge with analyzing these results is the absence of a universally accepted definition of delay. Even within each study, there was a wide variation in delay reporting and in the delay interval among the groups being compared. For example, the time intervals studied in the delayed intervention group for Ahmad et al., Warlick et al., Filippou et al., Cooperberg et al., and Dall’Era et al. were 31, 26.5, 20, 19.5, and 18 months, respectively. Despite these long delays, the number of patients in these groups was too small to affect the overall median delay. Furthermore, several studies suggested that patients with delays actually had improved outcomes, or had documented evidence that their institutional policy favored earlier treatment of patients with more advanced disease than those with lower risk disease.

#### Studies with low-, intermediate-, or high-risk prostate cancer exclusively

Seven of the 39 studies were limited only to low-risk patients (Supplementary Appendix A), whereas six studies included low- and intermediate-risk patients (Supplementary Appendix B). Several general observations concerning these data can be made. Studies with low-risk patients only (*n* = 38,915) had an average median delay of ∼4 months, were all in the setting of RP, and generally showed that delays as long as 6 months are likely to be safe. Longer delays might be safe for oncologic outcomes as shown by Warlick et al., van den Bergh et al., and Dall’Era et al.. However, some studies showed that delays longer than 6–12 months (Freedland et al., O’brien et al., Weiner et al., and Sun et al.) may negatively affect oncologic (adverse pathological features) and functional (sexual function and incontinence rates) outcomes. Studies that included low- and intermediate-risk patients (*n* = 13,210) had a median delay of 6.5 months (excluding two studies with missing median delay data), were all in the setting of RP, and also had mixed results. Four studies compared ∼3 vs. ∼19–31 months without any statistical difference in oncologic and pathologic outcomes (Holmstrom et al., Cooperberg et al., Filippou et al., and Ahmad et al.). On the other hand, other studies reported that delays >6–12 months were associated with worse pathological findings, worse biochemical recurrence, and increased need for post-operative RT (but not necessarily prostate cancer mortality). These adverse outcomes were likely driven by the intermediate-risk subgroup. One study exclusively included patients with high-risk disease treated with RP and concluded that delays >12 weeks were safe although patients in the >12 weeks group had more favorable characteristics (Supplementary Appendix C).

#### Studies with mixed-risk prostate cancer patients

The remaining 25 (64%) studies included combinations of low-, intermediate-, and high-risk patients (*n* = 1,110,647) (Supplementary Appendix D). There appears to be consistent evidence that lengthy delays are associated with worse outcomes in high-risk patients (Nguyen et al., Berg et al., Fossati et al., Zanaty et al., and Awasthi et al.). Papers that did not confirm this observation either had very short delays and/or explicitly excluded patients with long delays (e.g. >1 year). In addition, they included only favorable-risk patients, had a practice pattern that explicitly prioritized treatment of high-risk patients first, were underpowered, or included heterogenous cohorts. Three of the definitive RT papers explicitly favored short delays for high-risk patients and in one of them, nearly 75% of the patients with delays exceeding 3.3 months received ADT (Dong et al., Kwan et al., and Andrews el al.). The remaining RT study excluded men receiving ADT and supported the notion that delays compromise outcomes for high-risk prostate cancer (Nguyen et al.). For high-risk patients who received RP, a threshold of 6–12 months of delay or more appears to be associated with worsened outcomes.

General conclusions can also be reached concerning the 17 studies with mixed cohorts, after excluding the series in which the investigators noted possible institutional preference for treating high-risk patients earlier (Khan et al., Graefen et al., Andrews et al., Kwan et al., Hirasawa et al., O’Callaghan et al., Gupta et al., and Diamand et al.). Among those studies that failed to show an association with delay, two are small in cohort size and underpowered (Shibata et al. and Lee et al.), one excluded patients delayed >6 months (Aas et al.), two excluded patients delayed >1 year (Boorjian et al., and Vickers et al.), and four had very short median delay of <2 months (Lee at al., Phillips et al., Korets et al., and Khorana et al.). In another study which also did not find a difference with prolonged delay for high-risk patients, the percentage of patients with a delay >6 months was only ∼3% (Ginsburg et al.). In addition to statistical concerns of multicollinearity and overfitting, the percentage of patients with high Gleason grade group was also relatively small (∼25%), indicating selection bias and low power. Of the remaining 8 series not suffering from these limitations, six concluded that delays adversely impacted outcomes particularly for high-risk patients (Nam et al., Nguyen et al., Berg et al., Fossati et al., Zanaty et al., and Awasthi et al.). Thus, in adequately powered studies, not including ADT, most studies suggest that delays beyond 6–12 months adversely impact patients outcomes for patients with high-risk prostate cancer.

## Discussion

Several recommendations have been published during the COVID-19 pandemic on recommended treatment initiation delays for patients with prostate cancer, without deeply assessing the evidence behind such recommendations. While the COVID-19 pandemic is over, physicians are always asked about acceptable and safe definitive treatment delays due to vacations, logistical concerns, or psychosocial reasons. Our goal was to perform a narrative review of the literature on the available evidence of the impact of delays in the definitive treatment of prostate cancer, using RCTs and retrospective studies. The first category of RCT data likely provides an upper limit for estimating “reasonable delays”, while the retrospective post hoc studies may represent a “real world” reflection as what usually happens in the course of common practice. Our conclusions concerning these combined data are as follows. First, due to the nature of the data (variability in how data were collected, sub-categories assessed, variability of follow-up, the definition for delay used and endpoints) and the absence of any trials specifically designed to answer the question of delayed radical interventions in prostate cancer, it became clear that the available data were not suitable for a meta-analysis. Second, given the inherent bias in the studies available, it was not possible to assess bias in this narrative review. Third, although there appeared to be conflicting results between several series, the vast majority of the differences between studies can be explained. Fourth, it is very important to note that the best available evidence suggests that systemic treatment alone is inadequate and that “radical” local treatment (RP or RT) should not be omitted for younger patients with locally advanced disease. Finally, some general and safe conclusions concerning acceptable duration of delays can be made (see below and Table 2).

**Table 2:** Summary of general treatment guidelines.

	Clinical/pathologic features	Anticipated delay < 6 Months	Anticipated delay > 6 months
**Localized prostate cancer risk classes**			
Very low/low	Has the following: • T1–T2a • Grade Group 1 • PSA < 10 ng/mL	No intervention	Active surveillance Consider PSA at 6-month intervals Delays up to 7 years may be safe
Favorable intermediate	• No high-risk group features, has single intermediate risk factor: T2b–T2c or Grade Group 2 or PSA 10–20 ng/mL	No intervention	Active surveillance Consider PSA at 6-month intervals MRI may be used for staging to avoid misclassification
Unfavorable intermediate	• No high-risk group features, has two or more intermediate risk factors: T2b–T2c or Grade Group 2 or PSA 10–20 ng/mL OR • Grade group 3 OR • ≥50% biopsy cores positive	Treatment can probably be delayed 3–6 months safely	With ADT, delays of up to 8 months appear to be safe
High to very high	Has at least one high-risk feature: • T3a OR • Grade Group 4 or Grade Group 5 OR • PSA > 20 ng/mL	Treatment can probably be safely delayed 3 months but consider initiation of ADT if beyond 3 months	Treatment delays of up to 8 months are probably safe with ADT
**Post-prostatectomy**			
Adjuvant radiotherapy	For patients with high-risk features including pT3 disease, positive surgical margins, and/or node positive disease but undetectable PSA post-prostatectomy	Strongly consider early salvage (at PSA 0.10-0.2) rather than adjuvant radiotherapy especially for patients with limited high-risk features.	Strongly consider early salvage (at PSA 0.10-0.2) rather than adjuvant radiotherapy especially for patients with limited high-risk features.
Salvage radiotherapy	For patients with detectable and increasing PSA post-prostatectomy	Delaying radiotherapy is less well studied in the salvage setting. Decisions for delay should be made on a case-by-case basis. Consider PSA level and PSA doubling time. Consider ADT to delay radiation initiation.	Delaying radiotherapy is less well studied in the salvage setting. Decisions for delay should be made on a case-by-case basis. Consider PSA level and PSA doubling time. Consider ADT to delay radiation initiation.

*PSA* Prostate specific antigen, *ADT* androgen deprivation therapy, *RP* radical prostatectomy, *MRI* magnetic resonance imaging.

In light of presented data, patients and physicians may want to consider selectively and safely delaying the treatment of localized prostate cancer, if needed. Fortunately, there appears to be some available evidence based on which to make recommendations concerning the safety of such delays (Table 2). All patients with clinically localized prostate cancer should be reassured that delaying initiation of treatment (whether surgery or radiation) for 3–6 months is likely to be very safe. For patients with low-risk disease, delays measured in years is likely to be safe. For patients with unfavorable or high-risk disease (especially those considering radiation), ADT can be initiated and the neoadjuvant period can be extended to 8 months prior to the initiation of radiation, without compromising oncologic outcomes. In between these two groups (i.e. low- and unfavorable intermediate-risk/high-risk) are patients for whom the use of ADT may not appropriate (e.g. surgical patients, favorable intermediate-risk patients). For this subset of patients, delays should be individualized as some may behave more like the former group and others like the latter group. In the post-operative setting, salvage RT should replace adjuvant RT. For patients with PSA failure, impact of delaying salvage RT is less understood but NHT can be used to delay RT initiation.

The COVID-19 pandemic caused significant delays in prostate cancer screening and treatment initiation [46], leading to the publication of several guidelines on appropriate delays of definitive treatments. The conclusions from our analysis are similar to what others have published. In a systematic review published by Nguyen et al. in 2021, the authors concluded similarly that for patients with intermediate- and high-risk prostate cancer, treatment can be delayed up to 3 months without consequences, but delays beyond 6–9 months may increase risk of recurrence and worse pathological outcomes [47]. All the 24 included studies were retrospective. On the contrary, our analysis had 26 RCT and 39 retrospective study. In another study by Zaorsky et al. [48], the authors also similarly concluded that “treatment can be avoided or delayed until safe for very low, low, and favorable intermediate-risk disease and up to 4–6 months with NHT for unfavorable intermediate-risk, high-risk, and recurrence post-surgery”. To our knowledge, this was the first paper recommending management strategies for prostate cancer during the pandemic, and understandably given the urgency of the matter at that time, a detailed discussion for the bases of these recommendations was not available. Reasonable variations of generally the same conclusions were also provided by others as well [49, 50].

There are few important limitations that must be acknowledged. First, patients included on RCTs may be healthier than men in the general population. If this were true, we might be overestimating the benefits of delayed treatment because of competing risks, thus our estimates may be overly conservative. Another possible limitation is that with newer drugs and better imaging, progression could be detected earlier and patients with stable disease now requiring interventions could actually be spared definitive local treatment longer. In both of these scenarios, we would consider this more acceptable than asserting that an “unsafe” delay as being a “safe” delay. Thus, it appears that modest delays measured in weeks, months, and years for high-, intermediate-, and low-risk prostate cancer, respectively, appears to have relatively little impact on long-term outcomes. Furthermore, while we spoke about NHT or active surveillance as means to delay “definitive treatments”, we acknowledge their significance in the management of prostate cancer. NHT, for example, is central in the radiotherapy management paradigm and its use, whether in the clinical trials that justified its inclusion or in daily clinical practice, was not originally designed to “delay” definitive treatments but rather to improve outcomes. Our goal, however, for this narrative review was mainly to give patients a rough estimate of a period in months or years which is deemed acceptable to specifically postpone surgery or radiation even if it entails other interventions such as NHT.

We are hopeful that the summary of the available data presented here will help facilitate transparent communication to patients and their families as well as primary care physicians as they consider making referrals and evidence-based recommendations concerning treatment delays in men with clinically localized prostate cancer. Given the unlikely possibility that clinical trials will be designed to answer this question, we hope this discussion clarifies the reasoning behind the recommendations for acceptable durations of treatment delays in patients with prostate cancer.

## Supplementary information

Appendices A and B

## References

[1] Lazzeri G, Troiano G, Porchia BR, Centauri F, Mezzatesta V, Presicce G, et al. Waiting times for prostate cancer: A review. J Public Health Res. 2020;9:1778. 32550222 10.4081/jphr.2020.1778 PMC7282316

[2] Neal RD, Allgar VL. Sociodemographic factors and delays in the diagnosis of six cancers: analysis of data from the “National Survey of NHS Patients: Cancer”. Br J Cancer. 2005;92:1971–5. 15900296 10.1038/sj.bjc.6602623 PMC2361785

[3] Pattenden TA, Samaranayke D, Morton A, Ong WL, Murphy DG, Pritchard E, et al. Modern Active Surveillance in Prostate Cancer: A Narrative Review. Clin Genitourin Cancer. 2023;21:115–23. 36443163 10.1016/j.clgc.2022.09.003

[4] Care of Prostate Cancer Patients During the COVID-19 Pandemic: Recommendations of the NCCN. https://www.nccn.org/covid-19/default.aspx.

[5] Dell’Oglio P, Cacciamani GE, Muttin F, Mirabella G, Secco S, Roscigno M et al. Applicability of COVID-19 Pandemic Recommendations for Urology Practice: Data from Three Major Italian Hot Spots (BreBeMi). Eur Urol Open Sci. 2021;26:1–9. 10.1016/j.euros.2021.01.012 PMC7846227 33554150

[6] Yu J, Ouyang W, Chua MLK, Xie C. SARS-CoV-2 Transmission in Patients With Cancer at a Tertiary Care Hospital in Wuhan, China. JAMA Oncol. 2020;6:1108–10. 32211820 10.1001/jamaoncol.2020.0980 PMC7097836

[7] Siegel RL, Miller KD, Jemal A. Cancer statistics, 2019. CA Cancer J Clin. 2019;69:7–34. 30620402 10.3322/caac.21551

[8] Pernar CH, Ebot EM, Wilson KM, Mucci LA. The Epidemiology of Prostate Cancer. Cold Spring Harb Perspect Med. 2018;8:a030361. 29311132 10.1101/cshperspect.a030361 PMC6280714

[9] Siegel DA, O’Neil ME, Richards TB, Dowling NF, Weir HK. Prostate Cancer Incidence and Survival, by Stage and Race/Ethnicity - United States, 2001-2017. MMWR Morb Mortal Wkly Rep. 2020;69:1473–80. 33056955 10.15585/mmwr.mm6941a1 PMC7561091

[10] Emanuel EJ, Persad G, Upshur R, Thome B, Parker M, Glickman A, et al. Fair Allocation of Scarce Medical Resources in the Time of Covid-19. N Engl J Med. 2020;382:2049–55. 32202722 10.1056/NEJMsb2005114

[11] Zaorsky NG, Yu JB, McBride SM, Dess RT, Jackson WC, Mahal BA, et al. Prostate Cancer Radiotherapy Recommendations in Response to COVID-19. Adv Radiat Oncol. 2020;5:659–65. 32292839 10.1016/j.adro.2020.03.010 PMC7118610

[12] Tandogdu Z, Collins J, Shaw G, Rohn J, Koves B, Sachdeva A, et al. Management of patients who opt for radical prostatectomy during the COVID-19 pandemic: An International Accelerated Consensus Statement. BJU Int. 2020;127:729–41. 10.1111/bju.15299 33185026

[13] Roach M 3rd, Thomas K. Overview of randomized controlled treatment trials for clinically localized prostate cancer: implications for active surveillance and the United States preventative task force report on screening? J Natl Cancer Inst Monogr. 2012;2012:221–9. 23271777 10.1093/jncimonographs/lgs039 PMC3540886

[14] van den Bergh RC, Albertsen PC, Bangma CH, Freedland SJ, Graefen M, Vickers A, et al. Timing of curative treatment for prostate cancer: a systematic review. Eur Urol. 2013;64:204–15. 23453419 10.1016/j.eururo.2013.02.024 PMC3784981

[15] D’Amico AV, Manola J, Loffredo M, Renshaw AA, DellaCroce A, Kantoff PW. 6-month androgen suppression plus radiation therapy vs radiation therapy alone for patients with clinically localized prostate cancer: a randomized controlled trial. JAMA. 2004;292:821–7. 15315996 10.1001/jama.292.7.821

[16] Roach M 3rd, Bae K, Speight J, Wolkov HB, Rubin P, et al. Short-term neoadjuvant androgen deprivation therapy and external-beam radiotherapy for locally advanced prostate cancer: long-term results of RTOG 8610. J Clin Oncol. 2008;26:585–91. 18172188 10.1200/JCO.2007.13.9881

[17] Crook J, Ludgate C, Malone S, Perry G, Eapen L, Bowen J, et al. Final report of multicenter Canadian Phase III randomized trial of 3 versus 8 months of neoadjuvant androgen deprivation therapy before conventional-dose radiotherapy for clinically localized prostate cancer. Int J Radiat Oncol Biol Phys. 2009;73:327–33. 18707821 10.1016/j.ijrobp.2008.04.075

[18] Armstrong JG, Gillham CM, Dunne MT, Fitzpatrick DA, Finn MA, Cannon ME, et al. A Randomized Trial (Irish Clinical Oncology Research Group 97-01) Comparing Short Versus Protracted Neoadjuvant Hormonal Therapy Before Radiotherapy for Localized Prostate Cancer. Int J Radiat Oncol Biol Phys. 2010;81:35–45. 10.1016/j.ijrobp.2010.04.065 20797824

[19] Denham JW, Steigler A, Lamb DS, Joseph D, Turner S, Matthews J, et al. Short-term neoadjuvant androgen deprivation and radiotherapy for locally advanced prostate cancer: 10-year data from the TROG 96.01 randomised trial. Lancet Oncol. 2011;12:451–9. 21440505 10.1016/S1470-2045(11)70063-8

[20] Jones CU, Hunt D, McGowan DG, Amin MB, Chetner MP, Bruner DW, et al. Radiotherapy and short-term androgen deprivation for localized prostate cancer. N Engl J Med. 2011;365:107–18. 21751904 10.1056/NEJMoa1012348

[21] Pisansky TM, Hunt D, Gomella LG, Amin MB, Balogh AG, Chinn DM, et al. Duration of androgen suppression before radiotherapy for localized prostate cancer: radiation therapy oncology group randomized clinical trial 9910. J Clin Oncol. 2015;33:332–9. 25534388 10.1200/JCO.2014.58.0662 PMC4302214

[22] Malone S, Roy S, Eapen L, E C, MacRae R, Perry G, et al. Sequencing of Androgen-Deprivation Therapy With External-Beam Radiotherapy in Localized Prostate Cancer: A Phase III Randomized Controlled Trial. J Clin Oncol. 2020;38:593–601. 31829912 10.1200/JCO.19.01904 PMC7186584

[23] VanPoppel H, Ridder DD, Elgamal AA, DeVorrde WV, Werbrouck P, Ackaert K, et al. Neoadjuvant hormonal therapy before radical prostatectomy decreases the number of positive surgical margins in stage T2 prostate cancer: Interim results of a prospective randomized trial. J Urol. 1995;154:429–34. 7541860 10.1097/00005392-199508000-00027

[24] Homma Y, Akaza H, Okada K, Yokoyama M, Moriyama N, Usami M, et al. Preoperative endocrine therapy for clinical stage A2, B, and C prostate cancer: an interim report on short-term effects. Prostate Cancer Study Group. Int J Urol. 1997;4:144–51. 9179687 10.1111/j.1442-2042.1997.tb00161.x

[25] Aus G, Abrahamsson PA, Ahlgren G, Hugosson J, Lundberg S, Schain M, et al. Hormonal treatment before radical prostatectomy: a 3-year followup. J Urol. 1998;159:2013–6. discussion 6-7 9598509 10.1016/S0022-5347(01)63230-0

[26] Klotz LH, Goldenberg SL, Jewett M, Barkin J, Chetner M, Fradet Y, et al. CUOG randomized trial of neoadjuvant androgen ablation before radical prostatectomy: 36-month post-treatment PSA results. Canadian Urologic Oncology Group. Urology. 1999;53:757–63. 10197852 10.1016/s0090-4295(98)00616-5

[27] Schulman CC, Debruyne FM, Forster G, Selvaggi FP, Zlotta AR, Witjes WP. 4-Year follow-up results of a European prospective randomized study on neoadjuvant hormonal therapy prior to radical prostatectomy in T2-3N0M0 prostate cancer. European Study Group on Neoadjuvant Treatment of Prostate Cancer. Eur Urol. 2000;38:706–13. 11111188 10.1159/000020366

[28] Soloway MS, Pareek K, Sharifi R, Wajsman Z, McLeod D, Wood DP, et al. Neoadjuvant androgen ablation before radical prostatectomy in cT2bNxMo prostate cancer: 5-year results. J Urol. 2002;167:112–6. 11743286

[29] Widmark A, Klepp O, Solberg A, Damber JE, Angelsen A, Fransson P, et al. Endocrine treatment, with or without radiotherapy, in locally advanced prostate cancer (SPCG-7/SFUO-3): an open randomised phase III trial. Lancet. 2009;373:301–8. 19091394 10.1016/S0140-6736(08)61815-2

[30] Warde P, Mason M, Ding K, Kirkbride P, Brundage M, Cowan R, et al. Combined androgen deprivation therapy and radiation therapy for locally advanced prostate cancer: a randomised, phase 3 trial. Lancet. 2011;378:2104–11. 10.1016/S0140-6736(11)61095-7 PMC3243932 22056152

[31] Bill-Axelson A, Holmberg L, Garmo H, Taari K, Busch C, Nordling S, et al. Radical Prostatectomy or Watchful Waiting in Prostate Cancer - 29-Year Follow-up. N Engl J Med. 2018;379:2319–29. 30575473 10.1056/NEJMoa1807801

[32] Wilt TJ, Vo TN, Langsetmo L, Dahm P, Wheeler T, Aronson WJ, et al. Radical Prostatectomy or Observation for Clinically Localized Prostate Cancer: Extended Follow-up of the Prostate Cancer Intervention Versus Observation Trial (PIVOT). Eur Urol. 2020;77:713–24. 32089359 10.1016/j.eururo.2020.02.009

[33] Hamdy FC, Donovan JL, Lane JA, Mason M, Metcalfe C, Holding P, et al. 10-Year Outcomes after Monitoring, Surgery, or Radiotherapy for Localized Prostate Cancer. N Engl J Med. 2016;375:1415–24. 27626136 10.1056/NEJMoa1606220

[34] Thompson IM, Tangen CM, Paradelo J, Lucia MS, Miller G, Troyer D, et al. Adjuvant radiotherapy for pathological T3N0M0 prostate cancer significantly reduces risk of metastases and improves survival: long-term followup of a randomized clinical trial. J Urol. 2009;181:956–62. 19167731 10.1016/j.juro.2008.11.032 PMC3510761

[35] Wiegel T, Bottke D, Steiner U, Siegmann A, Golz R, Storkel S, et al. Phase III postoperative adjuvant radiotherapy after radical prostatectomy compared with radical prostatectomy alone in pT3 prostate cancer with postoperative undetectable prostate-specific antigen: ARO 96-02/AUO AP 09/95. J Clin Oncol. 2009;27:2924–30. 19433689 10.1200/JCO.2008.18.9563

[36] Bolla M, van Poppel H, Tombal B, Vekemans K, Da Pozzo L, de Reijke TM, et al. Postoperative radiotherapy after radical prostatectomy for high-risk prostate cancer: long-term results of a randomised controlled trial (EORTC trial 22911). Lancet. 2012;380:2018–27. 23084481 10.1016/S0140-6736(12)61253-7

[37] Hackman G, Taari K, Tammela TL, Matikainen M, Kouri M, Joensuu T, et al. Randomised Trial of Adjuvant Radiotherapy Following Radical Prostatectomy Versus Radical Prostatectomy Alone in Prostate Cancer Patients with Positive Margins or Extracapsular Extension. Eur Urol. 2019;76:586–95. 31375279 10.1016/j.eururo.2019.07.001

[38] Parker CC, Clarke NW, Cook AD, Kynaston HG, Petersen PM, Catton C, et al. Timing of radiotherapy after radical prostatectomy (RADICALS-RT): a randomised, controlled phase 3 trial. Lancet. 2020;396:1413–21. 33002429 10.1016/S0140-6736(20)31553-1 PMC7616947

[39] Kneebone A, Fraser-Browne C, Duchesne GM, Fisher R, Frydenberg M, Herschtal A, et al. Adjuvant radiotherapy versus early salvage radiotherapy following radical prostatectomy (TROG 08.03/ANZUP RAVES): a randomised, controlled, phase 3, non-inferiority trial. Lancet Oncol. 2020;21:1331–40. 33002437 10.1016/S1470-2045(20)30456-3

[40] Sargos P, Chabaud S, Latorzeff I, Magne N, Benyoucef A, Supiot S, et al. Adjuvant radiotherapy versus early salvage radiotherapy plus short-term androgen deprivation therapy in men with localised prostate cancer after radical prostatectomy (GETUG-AFU 17): a randomised, phase 3 trial. Lancet Oncol. 2020;21:1341–52. 33002438 10.1016/S1470-2045(20)30454-X

[41] Neal DE, Metcalfe C, Donovan JL, Lane JA, Davis M, Young GJ, et al. Ten-year Mortality, Disease Progression, and Treatment-related Side Effects in Men with Localised Prostate Cancer from the ProtecT Randomised Controlled Trial According to Treatment Received. Eur Urol. 2020;77:320–30. 31771797 10.1016/j.eururo.2019.10.030

[42] Vale CL, Fisher D, Kneebone A, Parker C, Pearse M, Richaud P, et al. Adjuvant or early salvage radiotherapy for the treatment of localised and locally advanced prostate cancer: a prospectively planned systematic review and meta-analysis of aggregate data. Lancet. 2020;396:1422–31. 33002431 10.1016/S0140-6736(20)31952-8 PMC7611137

[43] Shipley WU, Seiferheld W, Lukka HR, Major PP, Heney NM, Grignon DJ, et al. Radiation with or without Antiandrogen Therapy in Recurrent Prostate Cancer. N Engl J Med. 2017;376:417–28. 28146658 10.1056/NEJMoa1607529 PMC5444881

[44] Pollack A, Karrison TG, Balogh AG, Low D, Bruner DW, Wefel JS, et al. Short Term Androgen Deprivation Therapy Without or With Pelvic Lymph Node Treatment Added to Prostate Bed Only Salvage Radiation Therapy: The NRG Oncology/RTOG 0534 SPPORT Trial. Int J Radiat Oncol Biol Phys. 2018;102:S1–278.

[45] Carrie C, Hasbini A, de Laroche G, Richaud P, Guerif S, Latorzeff I, et al. Salvage radiotherapy with or without short-term hormone therapy for rising prostate-specific antigen concentration after radical prostatectomy (GETUG-AFU 16): a randomised, multicentre, open-label phase 3 trial. Lancet Oncol. 2016;17:747–56. 27160475 10.1016/S1470-2045(16)00111-X

[46] Mostafavi Zadeh SM, Tajik F, Moradi Y, Kiani J, Ghods R, Madjd Z. Impact of COVID-19 pandemic on screening and diagnosis of patients with prostate cancer: a systematic review protocol. BMJ Open. 2022;12:e063748. 36028267 10.1136/bmjopen-2022-063748 PMC9421918

[47] Nguyen DD, Haeuser L, Paciotti M, Reitblat C, Cellini J, Lipsitz SR, et al. Systematic Review of Time to Definitive Treatment for Intermediate Risk and High Risk Prostate Cancer: Are Delays Associated with Worse Outcomes? J Urol. 2021;205:1263–74. 33443458 10.1097/JU.0000000000001601

[48] Zaorsky NG, Yu JB, McBride SM, Dess RT, Jackson WC, Mahal BA, et al. Prostate Cancer Radiation Therapy Recommendations in Response to COVID-19. Adv Radiat Oncol. 2020;5:659–65. 32292839 10.1016/j.adro.2020.03.010 PMC7118610

[49] Detti B, Ingrosso G, Becherini C, Lancia A, Olmetto E, Ali E, et al. Management of prostate cancer radiotherapy during the COVID-19 pandemic: A necessary paradigm change. Cancer Treat Res Commun. 2021;27:100331. 33581491 10.1016/j.ctarc.2021.100331 PMC7864785

[50] Obek C, Doganca T, Argun OB, Kural AR. Management of prostate cancer patients during COVID-19 pandemic. Prostate Cancer Prostatic Dis. 2020;23:398–406. 32690870 10.1038/s41391-020-0258-7 PMC7371779

