# Supplementary material for: Delayed definitive management of localized prostate cancer: what do we know?
Source: Prostate Cancer Prostatic Dis. 2024 Aug 11;28(2):280–7. doi: 10.1038/s41391-024-00876-2 (PMC12106089; doi:10.1038/s41391-024-00876-2)
Supplement: Supplementary file 1 — Appendices A and B [file 41391_2024_876_MOESM1_ESM.docx]

**Appendix:** Selected Retrospective Series on Treatment Delays for Men with Clinically Localized Prostate Cancer

| **Appendix A - Low-risk prostate cancer** | | | | | |
| --- | --- | --- | --- | --- | --- |
| **No.** | **Author (Year) [Reference]** | **Source / Country** | **Definition of Delay, Endpoint** | **Treatment, years (no.)** | **Conclusions and comments** |
| 1 | Freedland (2006) | SEARCH Database | MD~2.5 mo: <90 vs 91 to 180 vs 181 to 270 > 270 days; PSA failure | RP; 1988-2004 (n=895) | LR patients only. Delays up to 180 days safe, > 180 days associated with worse outcomes. |
| 2 | Warlick (2006) | J. Hopkins (USA) | MD unspecified: 26.5 mo. vs 3 mo; BCR | RP; 1995-2005 (n=178) | LR patients only. Delay did not compromise outcome. |
| 3 | van den Bergh (2010) | Netherlands | MD~9.9 mo: 6 vs 31 mo; adverse pathology and PSA failure | RP; 1995-  (n=227) | LR patients only. No statistical differences in outcome with delay. |
| 4 | Dall’Era (2011) | UCSF (USA) | MD unspecified: 18 vs <6 mo; adverse pathology | RP; 1996-2008 (n=1408) | LR patients only. No differences with delay. |
| 5 | O’brien (2011) | Multi-institutional (USA) | MD unspecified: < 6 (MD 2.4) vs > 6 (MD 15.6) mo; adverse pathology and PSA failure | RP; 1989-2009 (n=1,111) | LR patients only. Delays > 6 mo associated with worse outcomes including GS=7-10 and BCR, even on multi-variable analysis |
| 6 | Sun (2012) | SEER (USA) | MD=2 mo: <3 vs >3 mo; complications and cancer specific mortality | RP; 1995-2005 (n=17,153) | LR patients only. Delay did not compromise cancer outcomes BUT associated with increased risk of erectile dysfunction and incontinence; favorable patients tended to have more delays. |
| 7 | Weiner (2015) | NCDB (USA) | MD 2.5 mo: <6, 6-9, 9-12, >12 mo. adverse pathology | RP, 2010-2011, (n=17,943) | LR patients only. Delays OK up to 12 mo. Delay >12 mo increased risk of adverse path |

| **Appendix B - Low- and intermediate-risk prostate cancer** | | | | | |
| --- | --- | --- | --- | --- | --- |
| **Ref** | **Author (Year)** | **Source / Country** | **Definition of Delay, Endpoint** | **Treatment, years (no.)** | **Conclusions and comments** |
| 8 | Holmström (2010) | Sweden | MD unspecified: 3.5 vs 19.2 mo; adverse pathology and PCSM | RP; 1997-2002 (n=2,566) | LR and IR only. Delays had no impact on path or PCSM. |
| 9 | Cooperberg (2011) | UCSF (USA) | MD unspecified: 3 vs 19.5 mo; adverse pathology and PSA failure | RP; -2010 (n=466) | LR and IR only. Underpowered study but delays associated with non-statistically significant increases in grade on path, pT3 and ECE. Bottomline: 16 months may be too long |
| 10 | Abern (2013) | Multi-institutional (USA) | MD <3 mo: 0-3 vs 3-6 vs 6-9 vs >9 mo; adverse pathology and PSA failure | RP; 1988-2011 (n=1561) | LR and IR only. Delays > 9 mo for IR is associated with BCR and PCSM. |
| 11 | Filippou (2015) | UCSF (USA) | MD: <6 vs 20 mo; adverse pathology | RP; 1990-2014 (n=678) | LR and IR only. Delay had no impact on adverse pathology after matching for pretreatment characteristics. |
| 12 | Loeb (2016) | Sweden | MD=<1yr: < 1 vs 1-2 vs > 2 yrs; adverse pathology, salvage RT, PCSM | RP; 1997-2007 (n=7608) | LR and IR only. Delays are bad: RP >2 yrs post-diagnosis had a worse path and increased risk of salvage RT, but not PCSM |
| 13 | Ahmad (2020) | Princess Margaret, Canada | MD unspecified: <6 vs 31 mo; adverse pathology and PSA failure | RP; 1992-2015 (n=575) | LR and IR patients; Delays are not associated with adverse outcomes. |

| **Appendix C – High-risk prostate cancer** | | | | | |
| --- | --- | --- | --- | --- | --- |
| **No.** | **Author (Year) [Reference]** | **Source / Country** | **Definition of Delay, Endpoint** | **Treatment, years (no.)** | **Conclusions and comments** |
| 14 | Reichard (2019) | Multi-institutional (USA) | MD=2.3 mo: <8 vs 8-12 vs > 12 weeks; PSA failure and PCSM | RP; 2005-2015, (n=1392) | HR patients only. Delays safe, delays > 12 weeks more favorable pts. |

| **Appendix D – Mixed risk prostate cancer** | | | | | |
| --- | --- | --- | --- | --- | --- |
| **No.** | **Author (Year) [Reference]** | **Source / Country** | **Definition of Delay, Endpoint** | **Treatment, years (no.)** | **Conclusions and comments** |
| 15 | Nam (2003) | Canada | MD=2.3 mo (15-951 days): < 3 vs ≥3 mo; PSA failure and DM | RP; 1987-97 (n=645) | Delays are bad |
| 16 | Khan (2004) | J. Hopkins (USA) | MD~3.5 mo (91-120 days): < 60 vs 61-90 vs 91-121 vs 121-150 vs >151 days; PSA failure | RP; 1989-1994 (n=926) | Delay up to 6 months is acceptable. Lower risk pts had more delays (>151 days) |
| 17 | Graefen (2005) | Hamburg (Germany) | MD=1.8 mo (5 to 518 days); recurrence-free survival | RP; 1992-2000 (n=795) | Delays didn’t matter but institutional policy to treat pts w high grades sooner and fairly narrow wait ranges, short follow-up |
| 18 | Nguyen (2005) | Multi-institutional (USA) | MD=2.5 mo (~75 days); BNED ASTRO definition | RT no ADT; 1992-2001 (n=460) | Delays (<2.5 vs >2.5 mo) are bad for HR pts but not LR |
| 19 | Shibata (2005) | Stanford (USA) | MD=2.5 mo: <60 vs 61-90 vs 91-120 vs 121-180 vs >180 days; PSA failure | RP; ~1996 (n=151) | Delay not associated with PSA Failure but smallest study with heterogeneous populations |
| 20 | Boorijan (2005) | MSKCC (USA) | The mean (interquartile range) delay 2.3 (1.6–3.3) mo, used as a continuous variable and in 3 mo intervals; PSA recurrence | RP; 1987-2002 (n=3149) | Delays from Bx to RP not important “within a year of diagnosis, even for those considered to be at high risk”, excluded pts with > 12 mo. |
| 21 | Andrews (2005) | Fox Chase (USA) | MD=3.1 mo: < 3 vs 3-6 vs 6-9 vs > 9 months; OS, CSS, DM, FFBF | RT no ADT; 1981-2001 (n=1322) | Delay not an independent prognostic factor; trends support selection bias |
| 22 | Vickers (2006) | MSKCC / Baylor (USA) | MD<3 mo: <90 vs >90 days; PSA failure | RP; 1987-2002 (n=3149) | Delays did not affect outcome |
| 23 | Kwan (2006) | British Columbia, Canada | MD=3.7 mo: < or >3.7mo; PSA control. | RT no ADT; 1993-2001 (n=1024) | Pts with longer delay did better but were more favorable suggestion selection bias. Differences disappeared on multi-variable analysis. |
| 24 | Lee (2006) | Iowa (USA) | MD=1.9 mo: (14-378 days); BCR and post-op complications. | RP; 2001-2004 (n=169) | Short delays, small study with favorable patients, and no impact on outcome seen. |
| 25 | Phillips (2007) | Multi-institutional (USA) | MD=1.9 mo: <3 vs >3 mo; BCR | RT or RP (No ADT); 1991-2004 (n=393) | Delay of > 3 mo safe. |
| 26 | Korets (2012) | Columbia (USA) | MD=1.5 mo: <60 vs 61-90 vs > 90 days; adverse path, PCSM and PSA failure | RP; 1990-2009 (n=1568) | Delays > 60 days had no impact on adverse path or PSA failure. |
| 27 | Berg (2015) | Columbia (USA) | MD=2.1 mo:15 days multiples (15, 30 …180 days); adverse pathology | RP; 1990-2011 (n=2212) | Delays are bad: for LR short delays are ok, but not for HR |
| 28 | Dong (2016) | Fox Chase (USA) | MD=3.3 mo: <3 vs 3-6 vs 6-9 vs 9-24 mo. OS, DM, and BCR. | RT ADT allowed; 1989-2013 (n=4064) | Delays not an independent prognostic factor, similar for all 3 risk groups regardless of use of ADT |
| 29 | Fossati (2017) | Milan, Italy | MD=2.8 mo: Delay as a continuous variable; PSA Failure. | RP; 2006-2011 (n=2653) | Delay associated with PSA failure in HR pts, keep <12 mo for HR. Very small number of patients with >12 mo delay. |
| 30 | Hirasawa (2017) | Tokyo, Japan | MD=3.8 mo: <3 vs 3- 6 vs > 6 mo; BCR | RP; 2006-2015 (n=793) | Delay of >6 mo are safe. Pts >6 mo actually did slightly better (p=0.18) but only 11% had delay > 6 mo. |
| 31 | O'Callaghan (2017) | Australia | MD~3.7 mo: Quartiles, Q1:35, Q2:86, Q3:138, Q4:264 days; OS, PCSM | RP and RT; 1998-2013 (n=3140) | Delays appear to be safe but shorter delays associated with worse disease |
| 32 | Morini (2018) | Brazil | Mean delay=6.4 mo: <6 vs 6-12 vs >12 mo; adverse pathology and PSA failure | RP; 2006-2014, (n=908) | Delays are safe |
| 33 | Zanaty (2018) | Canada | MD=5.1 mo: delay as continuous variable; PSA failure | RP; 2006-2015 (n=619) | Delays impact BCR for HR pts. Mean delay 153 overall but 169, 150, and 125 days for LR, IR and HR, respectively. |
| 34 | Awasthi (2019) | Multi-institutional (USA) | MD=3 mo: <3 vs 3- 6 vs > 6 mo; BCR. | RP; 1987-2015 (n=1807) | Delays up to 6 mo are safe, beyond this BCR increases. Mostly (~75%) LR & IR pts. Greater delays with AA men. |
| 35 | Aas (2019) | Norway | MD=3.1 mo: 0-60 vs 61-90 vs 91-120 vs 121-180 days; PCSM, adverse pathology and PSA failure | RP; 2001-2010 (n=5163) | Delays up to 180 days safe. |
| 36 | Khorana (2019) | NCDB (USA) | MD=1.9 mo; OS | RP & RT; 2004-2013 (n=944,246) | Delays not associated with worse outcomes. Quality of care, GS, and PSA not accounted for. |
| 37 | Gupta (2019) | J. Hopkins (USA) | MD~2.5 mo: <3 vs 3- 6 mo; adverse pathology, adjuvant treatments and PSA failure | RP; 2005-2018 (n=2303) | Unfavorable IR and HR; Delays up to 6 months are not associated with adverse outcomes. Shorter delays for higher grade disease. |
| 38 | Ginsburg (2020) | NCDB (USA) | MD=3 mo: <3, 3-6, 6-9, 9-12 mo; adverse pathology and post-op treatments | RP; 2010-2016 (n=128,062) | IR and HR patients. No difference for IR nor HR including GG 4 and 5. Only 3% delayed for >6 months (25% only with GG4-5)- selection bias. |
| 39 | Diamand (2020) | Multi-institutional (Europe) | MD=3.3mo: <3 vs >3 mo; adverse pathology, adjuvant treatments and PSA failure | RP; 2012-2019 (n=926) | IR and HR; Delays are not associated with adverse outcomes. Almost all patients with delay <9 mo. Shorter delays for HR. |

MD (Median delay); Low risk (LR), Intermediate risk (IR), High risk (HR), patients (pts); mo (months); biochemically no evidence of disease (BNED) based on PSA (prostate specific antigen); ADT (androgen deprivation therapy); RT (radiation therapy); RP (radical prostatectomy); NPCR (National prostate cancer registry); NCDB (National Cancer DataBase); PCSM (prostate cancer specific mortality); Bx (Biopsy); AS (active surveillance), CR (clinical relapse); AA (African American); NCDB (National Cancer Database); BCR (biochemical recurrence); GG (Gleason grade group); GS (Gleason score).

1. Freedland SJ, Kane CJ, Amling CL, Aronson WJ, Presti JC, Jr., Terris MK, et al. Delay of radical prostatectomy and risk of biochemical progression in men with low risk prostate cancer. The Journal of urology. 2006;175(4):1298-302; discussion 302-3.

2. Warlick C, Trock BJ, Landis P, Epstein JI, Carter HB. Delayed versus immediate surgical intervention and prostate cancer outcome. Journal of the National Cancer Institute. 2006;98(5):355-7.

3. van den Bergh RC, Steyerberg EW, Khatami A, Aus G, Pihl CG, Wolters T, et al. Is delayed radical prostatectomy in men with low-risk screen-detected prostate cancer associated with a higher risk of unfavorable outcomes? Cancer. 2010;116(5):1281-90.

4. Dall'Era MA, Cowan JE, Simko J, Shinohara K, Davies B, Konety BR, et al. Surgical management after active surveillance for low-risk prostate cancer: pathological outcomes compared with men undergoing immediate treatment. BJU international. 2011;107(8):1232-7.

5. O'Brien D, Loeb S, Carvalhal GF, McGuire BB, Kan D, Hofer MD, et al. Delay of surgery in men with low risk prostate cancer. The Journal of urology. 2011;185(6):2143-7.

6. Sun M, Abdollah F, Hansen J, Trinh QD, Bianchi M, Tian Z, et al. Is a treatment delay in radical prostatectomy safe in individuals with low-risk prostate cancer? The journal of sexual medicine. 2012;9(11):2961-9.

7. Weiner AB, Patel SG, Eggener SE. Pathologic outcomes for low-risk prostate cancer after delayed radical prostatectomy in the United States. Urol Oncol-Semin Ori. 2015;33(4).

8. Holmstrom B, Holmberg E, Egevad L, Adolfsson J, Johansson JE, Hugosson J, et al. Outcome of primary versus deferred radical prostatectomy in the National Prostate Cancer Register of Sweden Follow-Up Study. The Journal of urology. 2010;184(4):1322-7.

9. Cooperberg MR, Cowan JE, Hilton JF, Reese AC, Zaid HB, Porten SP, et al. Outcomes of active surveillance for men with intermediate-risk prostate cancer. J Clin Oncol. 2011;29(2):228-34.

10. Abern MR, Aronson WJ, Terris MK, Kane CJ, Presti JC, Jr., Amling CL, et al. Delayed radical prostatectomy for intermediate-risk prostate cancer is associated with biochemical recurrence: possible implications for active surveillance from the SEARCH database. The Prostate. 2013;73(4):409-17.

11. Filippou P, Welty CJ, Cowan JE, Perez N, Shinohara K, Carroll PR. Immediate Versus Delayed Radical Prostatectomy: Updated Outcomes Following Active Surveillance of Prostate Cancer. European urology. 2015;68(3):458-63.

12. Loeb S, Folkvaljon Y, Robinson D, Makarov DV, Bratt O, Garmo H, et al. Immediate versus delayed prostatectomy: Nationwide population-based study (.). Scand J Urol. 2016;50(4):246-54.

13. Ahmad AE, Richard PO, Leão R, Hajiha M, Martin LJ, Komisarenko M, et al. Does Time Spent on Active Surveillance Adversely Affect the Pathological and Oncologic Outcomes in Patients Undergoing Delayed Radical Prostatectomy? The Journal of urology. 2020;204(3):476-82.

14. Reichard CA, Nyame YA, Sundi D, Tosoian J, Wilkins L, Alam R, et al. Does time from diagnosis to treatment of high- or very-high-risk prostate cancer affect outcome? BJU international. 2019;124(2):282-9.

15. Nam RK, Jewett MA, Krahn MD, Robinette MA, Tsihlias J, Toi A, et al. Delay in surgical therapy for clinically localized prostate cancer and biochemical recurrence after radical prostatectomy. The Canadian journal of urology. 2003;10(3):1891-8.

16. Khan MA, Mangold LA, Epstein JI, Boitnott JK, Walsh PC, Partin AW. Impact of surgical delay on long-term cancer control for clinically localized prostate cancer. The Journal of urology. 2004;172(5 Pt 1):1835-9.

17. Graefen M, Walz J, Chun KH, Schlomm T, Haese A, Huland H. Reasonable delay of surgical treatment in men with localized prostate cancer--impact on prognosis? European urology. 2005;47(6):756-60.

18. Nguyen PL, Whittington R, Koo S, Schultz D, Cote KB, Loffredo M, et al. The impact of a delay in initiating radiation therapy on prostate-specific antigen outcome for patients with clinically localized prostate carcinoma. Cancer. 2005;103(10):2053-9.

19. Shibata A, Mohanasundaram UM, Terris MK. Interval from prostate biopsy to radical prostatectomy: effect on PSA, Gleason sum, and risk of recurrence. Urology. 2005;66(4):808-13.

20. Boorjian SA, Bianco FJ, Jr., Scardino PT, Eastham JA. Does the time from biopsy to surgery affect biochemical recurrence after radical prostatectomy? BJU international. 2005;96(6):773-6.

21. Andrews SF, Horwitz EM, Feigenberg SJ, Eisenberg DF, Hanlon AL, Uzzo RG, et al. Does a delay in external beam radiation therapy after tissue diagnosis affect outcome for men with prostate carcinoma? Cancer. 2005;104(2):299-304.

22. Vickers AJ, Bianco FJ, Jr., Boorjian S, Scardino PT, Eastham JA. Does a delay between diagnosis and radical prostatectomy increase the risk of disease recurrence? Cancer. 2006;106(3):576-80.

23. Kwan W, Pickles T, Duncan G, Liu M, Paltiel C. Relationship between delay in radiotherapy and biochemical control in prostate cancer. International journal of radiation oncology, biology, physics. 2006;66(3):663-8.

24. Lee DK, Allareddy V, O'Donnell M A, Williams RD, Konety BR. Does the interval between prostate biopsy and radical prostatectomy affect the immediate postoperative outcome? BJU international. 2006;97(1):48-50.

25. Phillips JJ, Hall MC, Lee WR, Clark PE. Does a delay in initiating definitive therapy affect biochemical recurrence rates in men with clinically localized prostate cancer? Urologic oncology. 2007;25(3):196-200.

26. Korets R, Seager CM, Pitman MS, Hruby GW, Benson MC, McKiernan JM. Effect of delaying surgery on radical prostatectomy outcomes: a contemporary analysis. BJU international. 2012;110(2):211-6.

27. Berg WT, Danzig MR, Pak JS, Korets R, RoyChoudhury A, Hruby G, et al. Delay from biopsy to radical prostatectomy influences the rate of adverse pathologic outcomes. The Prostate. 2015;75(10):1085-91.

28. Dong Y, Li T, Churilla TM, Viterbo R, Sobczak ML, Smaldone MC, et al. Effects of Time to Treatment on Biochemical and Clinical Outcomes for Patients With Prostate Cancer Treated With Definitive Radiation. Clin Genitourin Cancer. 2016;14(5):e463-e8.

29. Fossati N, Rossi MS, Cucchiara V, Gandaglia G, Dell'Oglio P, Moschini M, et al. Evaluating the effect of time from prostate cancer diagnosis to radical prostatectomy on cancer control: Can surgery be postponed safely? Urologic oncology. 2017;35(4):150 e9- e15.

30. Hirasawa Y, Ohori M, Sugihara T, Hashimoto T, Satake N, Gondo T, et al. No clinical significance of the time interval between biopsy and robotic-assisted radical prostatectomy for patients with clinically localized prostate cancer on biochemical recurrence: a propensity score matching analysis. Japanese journal of clinical oncology. 2017;47(11):1083-9.

31. O'Callaghan ME, Shi Z, Kopsaftis T, Moretti K. Prostate cancer outcomes and delays in care. Int Urol Nephrol. 2017;49(3):449-55.

32. Morini MA, Muller RL, de Castro Junior PCB, de Souza RJ, Faria EF. Time between diagnosis and surgical treatment on pathological and clinical outcomes in prostate cancer: does it matter? World journal of urology. 2018;36(8):1225-31.

33. Zanaty M, Alnazari M, Ajib K, Lawson K, Azizi M, Rajih E, et al. Does surgical delay for radical prostatectomy affect biochemical recurrence? A retrospective analysis from a Canadian cohort. World journal of urology. 2018;36(1):1-6.

34. Awasthi S, Gerke T, Park JY, Asamoah FA, Williams VL, Fink AK, et al. Optimizing Time to Treatment to Achieve Durable Biochemical Disease Control after Surgery in Prostate Cancer: A Multi-Institutional Cohort Study. Cancer Epidemiol Biomarkers Prev. 2019;28(3):570-7.

35. Aas K, Fossa SD, Kvale R, Moller B, Myklebust TA, Vlatkovic L, et al. Is time from diagnosis to radical prostatectomy associated with oncological outcomes? World journal of urology. 2019;37(8):1571-80.

36. Khorana AA, Tullio K, Elson P, Pennell NA, Grobmyer SR, Kalady MF, et al. Time to initial cancer treatment in the United States and association with survival over time: An observational study. PloS one. 2019;14(3):e0213209.

37. Gupta N, Bivalacqua TJ, Han M, Gorin MA, Challacombe BJ, Partin AW, et al. Evaluating the impact of length of time from diagnosis to surgery in patients with unfavourable intermediate-risk to very-high-risk clinically localised prostate cancer. BJU international. 2019;124(2):268-74.

38. Ginsburg KB, Curtis GL, Timar RE, George AK, Cher ML. Delayed Radical Prostatectomy is Not Associated with Adverse Oncological Outcomes: Implications for Men Experiencing Surgical Delay Due to the COVID-19 Pandemic. The Journal of urology. 2020:101097JU0000000000001089.

39. Diamand R, Ploussard G, Roumiguie M, Oderda M, Benamran D, Fiard G, et al. Timing and delay of radical prostatectomy do not lead to adverse oncologic outcomes: results from a large European cohort at the times of COVID-19 pandemic. World journal of urology. 2020.
